# Addition of host genetic variants in a prediction rule for post meningitis hearing loss in childhood: a model updating study

**DOI:** 10.1186/1471-2334-13-340

**Published:** 2013-07-23

**Authors:** Marieke S Sanders, Rogier CJ de Jonge, Caroline B Terwee, Martijn W Heymans, Irene Koomen, Sander Ouburg, Lodewijk Spanjaard, Servaas A Morré, A Marceline van Furth

**Affiliations:** 1 Department of Pediatric Infectious Diseases - Immunology, and Rheumatology, VU University Medical Center, Amsterdam, The Netherlands; 2 Department of Medical Microbiology, Laboratory for Immunogenetics, VU University Medical Center, Amsterdam, The Netherlands; 3 Department of Neonatology, Erasmus MC - Sophia Children’s Hospital, Room Sp-3434, PO-box 2040, Rotterdam, 3000 CB The Netherlands; 4 Department of Epidemiology and Biostatistics and the EMGO Institute for Health and Care Research, VU University Medical Center, Amsterdam, The Netherlands; 5 Department of Pediatrics, Westfriesgasthuis, Hoorn, The Netherlands; 6 Department of Medical Microbiology, Netherlands Reference Laboratory for Bacterial Meningitis, Academic Medical Center, Amsterdam, The Netherlands; 7 Department of Genetics and Cell Biology, Institute for Public Health Genomics, Research Schools GROW and CAPHRI, Faculty of Health, Medicine & Life Sciences, University of Maastricht, Maastricht, The Netherlands

**Keywords:** Genetics, SNP, Risk, Prediction, Bacterial meningitis, Hearing loss, Child

## Abstract

**Background:**

Sensorineural hearing loss is the most common sequela in survivors of bacterial meningitis (BM). In the past we developed a validated prediction model to identify children at risk for post-meningitis hearing loss. It is known that host genetic variations, besides clinical factors, contribute to severity and outcome of BM. In this study it was determined whether host genetic risk factors improve the predictive abilities of an existing model regarding hearing loss after childhood BM.

**Methods:**

Four hundred and seventy-one Dutch Caucasian childhood BM were genotyped for 11 single nucleotide polymorphisms (SNPs) in seven different genes involved in pathogen recognition. Genetic data were added to the original clinical prediction model and performance of new models was compared to the original model by likelihood ratio tests and the area under the curve (AUC) of the receiver operating characteristic curves.

**Results:**

Addition of *TLR9*-1237 SNPs and the combination of *TLR2* + 2477 and *TLR4* + 896 SNPs improved the clinical prediction model, but not significantly (increase of AUC’s from 0.856 to 0.861 and from 0.856 to 0.875 (*p* = 0.570 and 0.335, respectively). Other SNPs analysed were not linked to hearing loss.

**Conclusions:**

Although addition of genetic risk factors did not significantly improve the clinical prediction model for post-meningitis hearing loss, AUC’s of the pre-existing model remain high after addition of genetic factors. Future studies should evaluate whether more combinations of SNPs in larger cohorts has an additional value to the existing prediction model for post meningitis hearing loss.

## Background

Bacterial meningitis (BM) is the leading cause of acquired hearing impairment in children [1]. The reported overall incidence of sensorineural hearing loss (HL) in children surviving BM ranges from 7-36% [2-5]. It is thought that the large differences in reported incidences is explained by underestimation in some studies due to the difficulties in detecting HL. Because in mostly audiometric testing is only performed in clinical suspected cases of HL, many cases are late or never diagnosed [5]. Especially in children, early identification and rehabilitation of HL is indispensable because even mild changes in hearing abilities may impair auditory, linguistic, communication and learning skills with life-long consequences. For that reason, routine hearing evaluation is recommended in the standard follow-up program of childhood BM aiming to achieve more timely intervention [6]. To support the recognition of patients at high risk for HL after BM, Koomen et al. developed a clinical prediction model based on five predictors, including: duration of symptoms prior to admission longer than two days, the absence of petechiae, cerebrospinal fluid (CSF) glucose level ≤0.6 mmol/L, *Streptococcus pneumoniae* as causative pathogen and the presence of ataxia during the illness [3]. With this rule children at risk for HL can be identified in an early stage of the disease. It was recently successfully validated in an independent validation cohort of childhood BM survivors [7]. Besides clinical, environmental and pathogen-related factors, the ability of the host’s innate immune system to clear bacterial infections also influences the course of BM. In meningitis caused by *Neisseria meningitidis* or *S. pneumoniae,* host genetic factors are shown to play an important role [8,9]. Single nucleotide polymorphisms (SNPs) in genes encoding for receptors involved in recognition of *S. pneumoniae* and *N. meningitidis* are associated with severity of both meningococcal and pneumococcal infections [10,11]. Mice studies have shown that Toll-like receptor (TLR) mediated signaling is important in the initiation of the inflammatory response in the central nervous system (CNS) during pneumococcal meningitis [12,13]. This is also shown in the cochlea, since in this same animal model TLR-associated adapter molecule Myd88 knockout mice developed significantly less HL and had diminished cochlear inflammation compared to wild type mice [14]. There is increasing evidence that TLRs contribute to cochlear damage in meningitis [15]. We recently found an association of SNPs in *TLR-2*, -4 and −9, with an increased risk of HL in survivors of childhood BM suggesting that SNPs in TLRs and other peptides involved in pathogen recognition may be valuable markers to predict the individual risk to develop post-meningitis HL [16].

The aim of this study was to determine whether addition of host genetic risk factors in the pathogen recognition system could improve the prediction model of post-BM HL compared to the prediction model using clinical risk factors alone in children with pneumococcal and meningococcal meningitis.

## Methods

### Study population and collection of clinical data

The cohort used in this study is composed of two independent, comparable cohorts of school-age BM survivors: a development cohort and a validation cohort, both described in detail in the original studies [3,7]. In short, patients and data in both cohorts were retrospectively selected from data on bacterial cerebrospinal fluid (CSF) isolates of the Netherlands Reference Laboratory for Bacterial Meningitis (NRLBM) of patients treated in 110 different Dutch hospitals. The NRLBM receives approximately 90% of the isolates of Dutch meningitis patients [17]. The diagnosis meningitis was based on the demonstration of pathogens or antigens of *S. pneumoniae* or *N. meningitidis* in the CSF by culture or latex agglutination respectively. Children with “complex onset” of meningitis (defined as meningitis secondary to immune deficiency states, cranial trauma, CNS surgery, and CSF shunt infections) or relapsing meningitis were excluded.

For construction of the development cohort, files of the NRLBM were searched for children born between January 1986 and December 1994 who survived BM between January 1990 and December 1995. Sixteen hundred and five children were eligible for inclusion and their pediatricians were approached to send the parents a letter requesting participation. Six hundred and twenty-eight were included, and their medical records were investigated for risk factors and for perceptive HL of >25 dB. After internal validation this model was transformed into a clinical prediction rule including the variables: duration of symptoms prior to admission longer than two days, the absence of petechiae, CSF glucose level ≤0.6 mmol/L, *S. pneumoniae* as causative pathogen and the presence of ataxia during the illness. With this rule, a total risk score was calculated for each patient. The risk scores and the matching probability of HL were visually presented in a nomogram for use in clinical practice [3].

The clinical prediction rule was successfully validated in the validation cohort consisting of 116 children. The cohort was constructed in 2005 from files of the NRLBM and consisted of children born between January 1993 and December 1999 who suffered from non-Hib BM between January 1997 and December 2001 (unpublished observations, de Jonge *et al.*).

For the present study, all Dutch-Caucasian survivors of BM caused by *S. pneumoniae* or *N. meningitidis* were selected from the combined development and validation cohorts. Parents (or guardians) of the patients were asked by mail to participate in the study and to return a sterile swab after collecting buccal DNA of the children.Genetic data for our study were collected in the period from 2006 till 2010. The Medical Ethical Committee of the VU University Medical Center approved this study. Information on HL was retrieved from medical records after parents’ permission. The outcome measure HL was defined as unilateral or bilateral perceptive loss of >25 dB and was based on findings in these records and on parental information provided in the questionnaires about the children’s health (Dutch versions of the CHQ and the HUI mark 2&3 [18,19]). Conductive HL was not included.

Information on hearing loss was also collected by reviewing medical records kept by the pediatrician and the otolaryngologist during admission and during follow-up.

### DNA isolation

DNA was isolated from the buccal swabs using the following procedure: after addition of 250 μl 10 mM Tris–HCl (pH 7.4) the sample was heated at 96 degrees Celsius for 10 minutes. After mixing for 10 seconds the swabs were removed and the sample was centrifuged (14,000 rpm).

### Genetic analysis

Genotyped SNPs include *TLR2*-16934 T > A (NCBI SNP CLUSTER ID: rs4696480), *TLR2* + 2477 G > A (rs5743708), *TLR4* + 896 A > G (rs4986790), *TLR9 -*1237 T > C (rs5743836) and *TLR9 +*2848 G > A (rs352140), nucleotide oligomerisation domain protein (*NOD)-1* + 32556 (T- > GG) (rs6958571), *NOD2* + 2209 C > T (rs2066844), *NOD2* + 2722 G > C (rs2066845), *NOD2* + 3020 ins C (rs5743293), Caspase (*CASP)-1* + 8404 A > G (rs2282659), and tumour necrosis factor-related apoptosis inducing ligand (*TRAIL)*-692 T > C (rs365238). Results of a selection of these SNPs were described before in previous studies in 393 patients [16]. DNA was genotyped by real-time PCR using the TaqMan AbiPrism® 7000 Sequence Detection System (Applied Biosystems, UK) with the standard TaqMan protocol and the LightCycler® 480 System (Roche Applied Science, US). Results were analyzed by two independent researchers.

SNPs within the same gene or in the same biological pathway that showed a significant or trend association with the outcome measure HL in univariable analysis were combined (described in detail later). Studied combinations of SNPs were: *TLR2*-16934, *TLR2* + 2477, and *TLR4 + 896* (stimulating MyD88 via TIRAP and triggering the intracellular signaling cascade), *TLR4 + 896*, *TLR9-1237* and *TLR9 + 2848* (activating the MyD88 pathway) and the three *NOD2* SNPs (+2209, +2722 and +3020) [20]. *TLR9* haplotypes were determined by genotyping of both *TLR9*-1237 T > C and *TLR9* + 2848 G > A, which allows 4 locus haplotypes to be distinguished, as described by Lazarus et al. [21].

### Statistics

Genetic variables were coded as categorical variables with the following assigned categories in single gene analysis: “0” = no mutant alleles, “1” = one mutant allele, “2” = two mutant alleles. In children with- and without HL the distributions of all 11 SNPs and *TLR9* haplotypes were compared. Univariable analysis was performed to explore associations of genetic variables with the outcome measure HL by χ^2^ tests. Fisher’s Exact test was used if the data did not meet the criteria for a valid χ^2^-test. SNPs that showed a significant association (p < 0.05)with HL were further explored to see whether combined carriage of two or more specific SNPs resulted in more significant associations. Combined genes were coded as categorical variables by specific codes: “0” = no mutant alleles in both genes, “1” and “2” = one of both specific mutant allele in both genes “3” = four mutant alleles in both genes. Statistical significance was considered with 2-tailed *p*-values of <0.05.

To investigate whether genetic single and combined variables were able to improve the predictive ability of the clinical model we separately selected the most important genetic predictors for HL by using the least absolute shrinkage and selection operator (Lasso) method [22]. This is a statistical method to reliably select variables when there are more variables compared to the outcome categories (also called the events per variable problem) [23]. In a subsequent step the incremental predictive value of the most important genetic variables selected with the lasso method was assessed. Each important genetic variable was added to the clinical prediction model and the log likelihood values of the models with and without the genetic variable were compared and tested for significance conducting likelihood ratio tests [22]. Furthermore, the discriminative ability was compared based on the AUC of the ROC [22,24]. AUC’s of the models were obtained and tested for significant differences by using bootstrapping techniques [25]. We also used reclassification tables to assess if subjects were reclassified to appropriate risk categories if genetic variables were added to the model. For this purpose the Net Reclassification Index (NRI) was calculated for different probability values of HL (ranging from 10% to 90%) [22,26].

Patients with missing data in one of the five predictors or SNPs were excluded from analysis. SNPs that could not be genotyped after 3 real-time PCR assays were also excluded from analysis.

For statistical analysis, SPSS Statistics 17.0 (IBM Corporation, Somers, NY) and R (The R Project for Statistical Computing) were used.

## Results

### Participants

After exclusion of the cases of meningitis caused by pathogens other than *N. meningitidis* and *S. pneumoniae* and non-Dutch-Caucasians from both cohorts, 669 patients were eligible. They were invited to participate in the study. A total of 471 (70%) returned an intact buccal swab and an informed consent form. Reasons why patients were not included were: refusal to participate (6%), no response (20%) and damage to the swabs during mail delivery (4%). Our cohort consisted of 391 meningococcal meningitis (MM) patients and 80 pneumococcal meningitis (PM) patients (n = 395 children from the development cohort and 76 children from the validation cohort).The mean age of the patients at infection was 2.6 years (range 0 – 9). Forty-five percent of the children were female and 55% were male. There was no significant difference in distribution of the five predictors of the prediction rule or SNP distribution between both cohorts. Table 1 provides an overview of patient characteristics and included clinical variables of the original model.

**Table 1:** Patients and clinical variables

**Characteristics**	**Study cohort *n*** **= 471**			
	**Total**	**(% missing)**	**Cases ( *n *)**	**(%)**
**General characteristics**				
Male gender ^a^	471	(0)	260	55.2%
**Outcome measure**				
Hearing loss ^a^	471	(0)	34	7.2%
**Clinical predictors**				
Duration of symptoms > 2 days^a^	464	(1.5)	110	23.7%
Petechiae ^a^	463	(1.7)	273	59.0%
CSF glucose ≤ 0.6 mmol/l ^a^	418	(11.2)	125	29.9%
Causative pathogen in CSF:	471	(0)		
*N. meningitidis*^a^			391	83%
*S. pneumoniae*^a^			80	17%
(transient) ataxia ^a. b^	471	(0)	16	3.4%

^a^ Number of subjects (%).

^b^ (Transient) ataxia was defined as signs of ataxia, which lasted at least until discharge from the hospital, as documented in the medical records.

Abbreviations:

*No.*: Number.

*CSF*: Cerebrospinal fluid.

### Genetic analysis

Table 2 provides an overview of genotype distributions of SNPs used in this study. A selection of these SNP distributions were described in previous studies [16,27,28].

**Table 2:** Genotype distributions of SNPs used in this study

**Genetic variables**	Total ^*^		**Wildtype**		**Heterozygous**		**Mutant**	
	N	%	N	%	n	%	n	%
*TLR2*-16934 T > A HL	32	94.1	7	21.9	15	46.9	10	31.3
*TLR2*-16934 T > A no HL	411	94.1	114	27.7	196	47.7	101	24.6
*TLR2* + 2477 G > A HL	34	100	34	100	0	0	0	0
*TLR2* + 2477 G > A no HL	430	98.4	382	88.8	46	10.7	2	0,5
*TLR4* + 896 A > G HL	34	100	25	73.5	8	23.5	1	2.9
*TLR4* + 896 A > G no HL	420	96.1	374	89.0	33	7.9	13	3.1
*TLR9*-1237 T > C HL	33	97.1	21	63.6	12	36.4	0	0
*TLR9*-1237 T > C no HL	430	98.4	320	74.4	101	23.5	9	2.1
*TLR9* + 2848 G > A HL	33	97.1	5	15.2	16	48.5	12	36.4
*TLR9* + 2848 G > A no HL	426	97.5	104	24.4	199	46.7	123	28.9
*NOD1* + 32556 T > GG HL	34	100	20	58.8	13	38.2	1	2.9
*NOD1* + 32556 T > GG no HL	414	94.7	239	57.7	148	35.7	27	6.5
*NOD2* +2209 C > T HL	34	100	32	94.1	2	5.9	0	0
*NOD2* +2209 C > T no HL	427	97.7	381	89.2	38	8.9	8	1.9
*NOD2* +2722 G > C HL	31	91.2	31	100	0	0	0	0
*NOD2* +2722 G > C no HL	421	96.3	410	97.4	8	1.9	3	0.7
*NOD2* +3020 ins C HL	33	97.1	31	93.9	2	6.1	0	0
*NOD2* +3020 ins C no HL	426	97.5	409	96.0	16	3.8	1	0.2
*CASP1* + 8404 A > G HL	34	100	22	64.7	10	29.4	2	5.9
*CASP1* + 8404 A > G no HL	433	99.11	258	59.6	145	33.5	30	6.9
*TRAIL*-692 T > C HL	33	97.1	26	78.8	6	18.2	1	3.0
*TRAIL*-692 T > C no HL	420	96.1	339	80.7	72	17.1	9	2.1

^*^Genotypes and percentage of all included cases.

Abbreviations:

*TLR:* Toll-like receptor.

*HL*: Hearing loss.

*NOD*: Nucleotide oligomerization domain protein.

*CASP*: Caspase.

*TRAIL*: Tumour necrosis factor related apoptosis inducing ligand.

### Missing data

Percentage of missing data in genetic variables ranged from 0.8% in *CASP1* + 8404 up to 5.9% in *TLR2*-16934. Percentage of missing data in clinical risk factors were 0% in “causing pathogen” and “ataxia”, 1.5% in “duration of symptoms > 2 days”, 1.7% in “petechiae” and highest in “CSF glucose” (11.3%). Patients with missing data were excluded from the models. This resulted in 14% missing cases in the clinical model and 28% of missing cases during the selection of genetic variables.

### Selection of genes by Lasso

The most important genetic variables selected by the Lasso method (and coefficients) were: *TLR4* recessive alleles (coefficient −0.031), *TLR9*-1237 dominant alleles (coefficient 0.372) and *NOD2SNP13* dominant alleles (coefficient 0.183) in the single gene analysis. Combinations of *TLR2* + 2477 and *TLR4* (coefficient 1.053), *TLR2*-19634 and *TLR4* (coefficient 0.124), TLR2 + 2477 and *TLR9* + 2848 (coefficient 0.974) were all included in the analysis.

### Addition of SNPs to the clinical prediction model

Results of the performance of the original clinical prediction model compared with that of different models extended with genetic variables selected by the lasso method are presented in Table 3. Likelihood ratio tests were performed to test the goodness of fit between the two models. The AUC curve of the original clinical model was 0.856. Addition of TLR4 SNPs to the clinical model resulted in a slightly decreased AUC. Addition of TLR9-1237 to the clinical model slightly increased the AUC curve to 0.861, though this was not significant (p = 0.570). NOD2 SNPs did not improve the clinical model.

**Table 3:** Results of the performance of the original clinical model compared with that of different models extended with genetic variables selected by the lasso method

**SNP / SNP combination**	**Log likelihood values**	**AUC of ROC**	**95% CI of AUC of ROC**	***p*-value**^**a**^
**Clinical model**		0.856	0.794-0.909	
***TLR4***	0.2802	0.854	0.786-0.911	0.780
***TLR9*-1237**	−0.5675	0.861	0.798-0.915	0.570
***NOD2*-*SNP13***	0.8338	0.855	0.796-0.908	0.404
***TLR2*** **+ 2477 and *TLR4***	−0.9646	0.875	0.816-0.925	0.335
***TLR2*-19634 and *TLR4***	−0.8836	0.869	0.812-0.920	0.377
***TLR2*** **+ 2477 and *TLR9*** **+ 2848**	0.0843	0.855	0.780-0.918	0.933

^a^ AUC combined SNP model versus clinical model.

Abbreviations:

*SNP*: Single nucleotide polymorphism.

*AUC*: Area Under the Curve.

*ROC*: Receiver Operator Curve.

*95% CI*: 95% confidence interval.

*TLR*: Toll-like receptor.

*NOD*: Nucleotide Oligomerization Domain protein.

The AUC was 0.875 after addition of the combination of TLR2 +2477 and TLR4 SNPs to the clinical model, which was not significant (p = 0.335). This was also observed after addition of TLR2-16934 and TLR4 (AUC 0.869, p = 0.377). Addition of neither the combination of TLR2 + 2477 and TLR9 + 2848, nor TLR9 genotypes did not significantly improve the AUC the model (results not shown).

**Figure 1:**
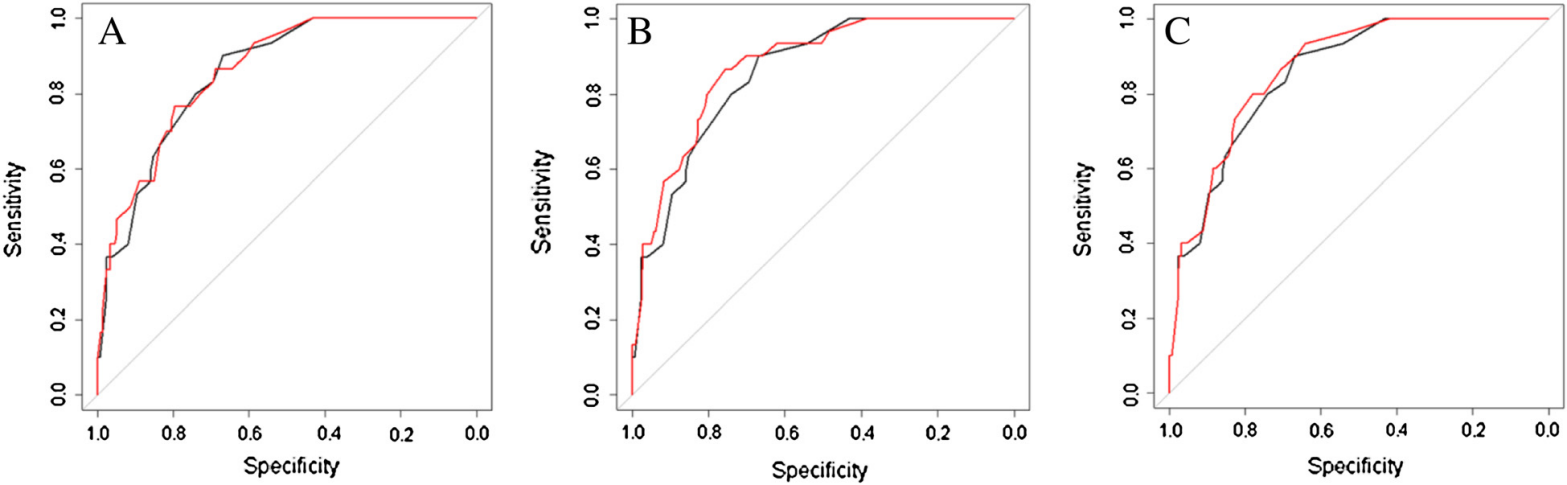
**ROC curves before and after addition of genetic variants that showed an improvement compared to the original model.** Black line: ROC curve of the original model (AUC 0.856). Red line: ROC curve of the original model including SNPs. **A**. Addition of *TLR9*-1237 to the original model (AUC 0.861). **B**. Addition of the combination of *TLR2* + 2477 and *TLR4* SNPs to the original model (AUC 0.875). **C**. Addition of the combination of *TLR2*-16934 and *TLR4* SNPs to the original model (AUC 0.869).

Figure 1 shows the AUC’s of the ROC’s of the original model compared to those of the new models including genetic variables that showed an increase in AUC.

Results of reclassification tables showed that addition of SNPs by different probabilities of HL did not improve the detection of cases and non-cases (data not shown).

## Discussion

In this study we determined whether host genetic risk factors improve the predictive abilities of an existing model regarding HL after BM in childhood. Although in univariable analysis *TLR* SNPs were significantly associated with HL [16], addition of these high risk genes did notresult in a significant improvement of a clinical prediction model for HL after BM.Using reclassification tables, a new technique for assessing the performance of prediction models, no improvement of the model was observed, conform the results of the AUC’s.

In order to explain mechanisms that may underlie the role of TLRs in post-meningitis HL, we focus on pathogenesis of meningitis. Bacteria spread from the subarachnoid space to the inner ear through the cochlear aqueduct, along the eighth nerve or the blood vessels of the blood-labyrinth barrier [15], inducing a suppurative labyrinthitis. As a result, the blood-labyrinth barrier and hair cells are damaged and neurons in the spiral ganglion show apoptosis. In the inner ear, bacteria multiply uncontrolled and after autolysis bacterial components are released, binding to pathogen recognition receptors (PRRs) present on immunocompetent endothelial cells and fibrocytes [29]. PRRs initiate the immune response and stimulate production of cytokines. There is increasing evidence for a role of TLRs in mediating cochlear damage in meningitis [15].

In general, a prediction model is interpreted to be excellent, good, or fair, when its AUC is 0.9 to 1.0, 0.8 to 0.9, or 0.7 to 0.8, respectively. The AUC of the clinical model was 0.856, thus it can be considered as a good model. Improving a model that is already considered as good, is difficult because only very strong predictors may result in a significant improvement, while other moderately strong predictors do not affect the model [30].

Our results are consistent with findings of other studies. Clinical factors, in contrast to genetic factors are frequently included in clinical prediction models for other diseases. For instance, numerous studies have investigated the predictive ability of genetic models in type II diabetes [31]. Almost without exception, the genetic risk models (including 18–40 SNPs) had lower AUC values than the clinical models. AUC values from genetic models ranged from 0.55 to 0.68 and those from clinical models from 0.61 to 0.92. Moreover, addition of genetic factors showed no or only marginally improved AUC beyond that of clinical risk models [31]. Recently two studies on predictive ability of SNPs in inflammatory diseases were published. In one study, addition of genetic risk factors to clinical predictors did not improve the prediction of risk of rheumatoid arthritis [32]. In another study, predictability of three knee osteoarthritis genes was poor (AUC 0.55 compared to 0.68 for clinical data only), but likelihood ratio improved slightly (AUC 0.69) by combining genes with clinical data. After age adjustment of controls, the combined AUC increased to 0.74 [33]. It should be mentioned that design and population characteristics were found to importantly affect the observed predictive performance of risk models [31,34]. In general and by definition, the predictive ability of risk models is higher when there are larger differences between cases and controls on the risk factors included in the risk model. In our study, population characteristics that may have negatively influenced the predictability of the model included age, sex and causative pathogen since these factors contribute to heterogeneity between groups which may differentially affect the outcome HL. The number of cases of HL was too small to divide our population in specific subgroups. Inclusion of a larger number of patients prospectively would enable to make these selections.

Although limited in the way mentioned above, we believe this study has some strengths. To our knowledge, the role of genetic pre-disposition to post-meningitis HL has only once been publicized earlier in a letter including 5 patients [35]. We are the first testing this hypothesis in a large patient group using very recently identified relevant genes [16]. The reclassification tables method was used since is it an upcoming, promising technique which may be of clinical relevance in the future [22,24]. For valid interpretation of genetic prediction studies it is crucial to optimize the quality of the reporting of these studies. In order to strengthen the reporting of Genetic Risk Prediction studies (GRIPS), a multidisciplinary workshop sponsored by the Human Genome Epidemiology Network developed a checklist of 25 items recommended [34]. Our study meets all these 25 items, pursuing a new standard of quality in genetic risk prediction research. We subscribe the vision of Goldstein that attention should shift from searching for common variants by genome scans of ever larger samples to studies of rare variants with a larger effect [36]. Using a candidate gene approach allows us to identify such genes with potential relevance in prediction.

It depends on the field of interest and the outcome measure whether biomarkers like SNPs have an additive value. For instance, for decision making in an emergency room only clinical predictors and biomarkers that are part of routine care and are easily available are useful. Though in oncology, where prognosis and decisions on therapy are often more a matter of days or weeks, genetic factors can be of great value. Currently, genetic factor analysis seems not contributory in the clinical context of BM. But, we are convinced that the concept of addition of genetic factors to clinical prediction rules is an interesting concept. Genetic risk factors will play an increasing key role in understanding pathophysiology of disease and outcome, and will become rapidly cheaper and faster available. To find the genetic factors with strongest predictive value new techniques like next-generation sequencing and genome-wide association studies (GWAS) can be used next to the candidate gene approach. Sequentially, the found genetic risk factors must be implemented in the development process of clinical prediction models to avoid the aforementioned problem difficult improvement of already strong models.

This study shows us the direction that studies including genetic predictors should be going. In order to use genetic factors in clinical practice several steps have to be taken. Our group is planning to develop a prediction model including both genetic and clinical data from the commencement of the study. It is more likely that genetic factors help to amend less robust prediction models requiring improvement. Other components of signal transduction routes of TLRs may be important in the pathogenesis of BM e.g. complement genes, signal transduction genes such as Toll/interleukin-1 receptor domain-containing adaptor protein (*TIRAP*) and cytokine genes. Inclusion of these SNPs and combinations in these analyses may strengthen the predictive abilities of new prediction models. Taken together, this will most likely lead to optimizing personalized public health programs and identification of high risk groups [37]. Complementary, health protection, fueled by genetic risk profiles will be a highly effective and efficient public health task [38]. In general, the success rate of timely translation of genome-based technologies to commercially feasible products or services with applicability in health care systems is significantly low. Lal *et al.* developed a new model of valorization to optimize integration of genome-based technologies into the healthcare system [39].

Another new method for early prediction of post meningitis hearing loss is the Gadolinium-enhanced MRI, reaching a sensitivity to 100% [40]. Since MRI is an expensive diagnostic tool, good prediction rules may help to select those children who need to undergo an MRI screening.

For further interpretation of the results of this study and more specific recommendations with regard to genetic predictors in meningitis, it is necessary to address our limitations. A disadvantage of using buccal DNA, which is taken by patients or parents themselves, is the potential poor quality of certain parts of DNA. For that reason, SNPs that could not be genotyped after 3 real-time PCR essays were excluded from analysis. We used a retrospective dataset which may induce selection bias and missing data. The definition of the outcome was based on documentation in patient records, and not based on a standardized protocol for HL. Further, the incidence of HL may be underestimated and the degree of hearing impairment is reported to be fluctuating [5,41]. Later deterioration of hearing in time after an initial absence of problems might occur. Last, we developed and validated the prediction model in a broad population with most possible pathogens included. It is known, and logically also found by the model, that HL is most common in pneumococcal BM. In our population the most common pathogen is *N. menigitidis*. With all changes in the distribution of responsible pathogens in recent years, due to vaccination programs and spontaneous decrease in incidence, it is reasonable to think the performance of the model changes in the nowadays situation. This again supports the need for continuing validation and redevelopment if necessary.

## Conclusion

We conclude that genetic factors did not increase the ability of the existing clinical model to predict the risk of post-meningitis HL significantly. Nevertheless, our study is new in showing the first results of the potential to combine genetic with clinical risk factors in BM. Knowledge about genetic risk factors may be used to target diagnostic, preventive, and therapeutic interventions for complex disorders based on a person’s genetic risk, or to complement existing risk models based on non-genetic factors [34]. Additional research including genetic variables from the commencement of the study, enforced by current technical advances in SNP detection is crucial to develop robust prediction rules ready for clinical practice.

## Abbreviations

AUC: Area under the curve; BM: Bacterial meningitis; CASP: Caspase; CHQ: Child health questionnaire; CNS: Central nervous system; CSF: Cerebrospinal fluid; HL: Hearing loss; HUI: Health utility index; MyD88: Myeloid differentiation primary response gene-88; NFκB: Nuclear factor kappa B; N. meningitidis: *Neisseria meningitidis*; NRLBM: Netherlands reference laboratory for bacterial meningitis; NOD: Nucleotide oligomerization domain protein; NRI: Net reclassification index; PCR: Polymerase chain reaction; PPR: Pathogen recognition receptor; ROC: Receiver operator curve; SNP: Single nucleotide polymorphism; S. pneumoniae: *Streptococcus pneumoniae*; TLR: Toll-like receptor; TRAIL: Tumor necrosis factor related apoptosis inducing ligand.

## Competing interests

The authors declare that they have no competing interests.

## Authors’ contributions

MS carried out the genetic studies, performed data collection and the statistics and drafted the manuscript. RJ performed data collection, the statistics and drafted the manuscript. CBT participated in the design of the study and advised in the statistical analysis. MWH performed the statistical analysis with regard to the prediction rule. IK developed the original model of the prediction model for hearing loss and helped to draft the manuscript. SO participated in the design and statistics of the study and coordination and helped to draft the manuscript. LS provided access to the files of the Netherlands Reference Laboratory for Bacterial Meningitis and helped to draft the manuscript. SAM supervised the study, participated in the design of the study and helped to draft the manuscript. AMvF supervised the study and helped to draft the manuscript. All authors read and approved the final manuscript.

## Authors’ information

Marieke S. Sanders and Rogier C.J. de Jonge are first authors.

## Pre-publication history

The pre-publication history for this paper can be accessed here:

http://www.biomedcentral.com/1471-2334/13/340/prepub
